# Single- and Multimodal Deep Learning of EEG and EDA Responses to Construction Noise: Performance and Ablation Analyses

**DOI:** 10.3390/s25216775

**Published:** 2025-11-05

**Authors:** Md Samdani Azad, Sungchan Lee, Minji Choi

**Affiliations:** 1 Department of Architectural Engineering, Inha University, Incheon 22212, Republic of Korea; samdaniazad@inha.ac.kr; 2 Division of Real Estate and Construction Engineering, Kangnam University, Yongin 16979, Republic of Korea; sclee@kangnam.ac.kr

**Keywords:** construction noise, annoyance detection, electroencephalography (EEG), electrodermal activity (EDA), sensors, convolutional neural networks (CNN), long short-term memory (LSTM)

## Abstract

The purpose of the study is to investigate human physiological responses to construction noise exposure using deep learning, applying electroencephalography (EEG) and electro-dermal activity (EDA) sensors. Construction noise is a pervasive occupational stressor that affects physiological states and impairs cognitive performance. EEG sensors capture neural activity related to perception and attention, and EDA reflects autonomic arousal and stress. In this study, twenty-five participants were exposed to impulsive noise from pile drivers and tonal noise from earth augers at three intensity levels (40, 60, and 80 dB), while EEG and EDA signals were recorded simultaneously. Convolutional neural networks (CNN) were utilized for EEG and long short-term memory networks (LSTM) for EDA. The results depict that EEG-based models consistently outperformed EDA-based models, establishing EEG as the dominant modality. In addition, decision-level fusion enhanced robustness across evaluation metrics by employing complementary information from EDA sensors. Ablation analyses presented that model performance was sensitive to design choices, with medium EEG windows (6 s), medium EDA windows (5–10 s), smaller batch sizes, and moderate weight decay yielding the most stable results. Further, retraining with ablation-informed hyperparameters confirmed that this configuration improved overall accuracy and maintained stable generalization across folds. The outcome of this study demonstrates the potential of deep learning to capture multimodal physiological responses when subjected to construction noise and emphasizes the critical role of modality-specific design and systematic hyperparameter optimization in achieving reliable annoyance detection.

## 1. Introduction

The widespread presence of noise in modern urban environments, especially the noise arising from construction activities, has become an increasingly recognized public health concern [1]. In contrast to many ambient noise sources that remain relatively constant, construction noise is characterized by pronounced fluctuations in intensity, irregular frequency profiles, and unpredictable temporal patterns. These features make it particularly disruptive and challenging for individuals to adapt to. Extended exposure to such noise has been consistently associated with heightened stress levels, sleep disturbances, cardiovascular strain, and cognitive impairments [2]. Experimental studies have demonstrated that construction noise contributes to elevated annoyance ratings, chronic stress, sleep disruptions, and increased blood pressure [3,4]. Beyond its physiological consequences, noise exposure impairs cognitive functions critical for effective daily functioning, such as sustained attention, concentration, and working memory. These impairments are particularly consequential in occupational contexts, where they can reduce decision-making capacity, increase error rates, and heighten accident risk on construction sites [5]. Recent investigations confirm these concerns: high-intensity construction noise has been linked to diminished hazard identification accuracy, slower reaction times, and altered cortical activation patterns indicative of reduced information-processing capacity [6,7]. These converging lines of evidence demonstrate that construction noise is not merely a nuisance but an occupational hazard with substantial implications for health, cognition, and safety.

Construction noise arises from a wide range of sources, including heavy equipment such as excavators and loaders, impact tools such as jackhammers and pile drivers, continuous engines such as compressors and generators, and specialized drilling or piling machinery. These noise sources produce acoustically diverse profiles that can be broadly classified into impulsive and tonal categories. Impulsive noises, often generated by jackhammers or nail guns, are characterized by sudden, high-amplitude bursts with rapid onset and short duration. They are typically perceived as startling and stressful, eliciting immediate autonomic arousal and heightened annoyance. Tonal noise, by contrast, is more continuous and frequency-specific, such as the low-frequency hum of a generator or the repetitive drilling sound of an earth auger. While less startling, they produce sustained annoyance, fatigue, and long-term psychological strain [8].

Psychophysiological signals offer a powerful means of capturing complex human responses to such stressors. Electroencephalography (EEG) provides insights into cortical activity related to attention, perception, and cognitive workload, while electrodermal activity (EDA) reflects sympathetic nervous system activation and emotional arousal [9,10]. Noise-induced changes in EEG are frequently observed as increases in frontal theta activity and decreases in alpha rhythms, patterns that indicate elevated cognitive effort and reduced relaxation [11,12,13,14]. EDA indices such as skin conductance level (SCL) and skin conductance responses (SCRs) rise in correspondence with heightened stress, anxiety, or attentional shifts [15]. Together, EEG and EDA provide complementary perspectives: EEG traces the neural processing of distraction and workload, while EDA captures autonomic arousal.

Traditional analyses of EEG and EDA have relied on hand-crafted features such as spectral band powers, event-related potential amplitudes, or counts of SCRs. While these approaches have generated valuable findings, they often fail to capture the nonlinear, high-dimensional nature of physiological responses in noisy environments. Deep learning has emerged as a transformative alternative by enabling hierarchical feature extraction directly from raw signals [16,17]. Convolution Neural Networks (CNNs) are particularly effective for EEG because they can capture local temporal patterns and spatial channel interactions, while long short-term memory (LSTM) is well-suited to EDA, which exhibits slower, sequential dependencies [18,19]. Recent advances in affective computing have shown that such models outperform traditional classifiers for detecting stress, fatigue, and emotional states from bio-signals [20,21,22,23]. Importantly, deep learning avoids the limitations of manual feature engineering, allowing models to uncover subtle and individualized physiological signatures of stress that might otherwise remain undetected.

A further step forward lies in multimodal fusion. Since EEG and EDA provide complementary information, integrating them allows for a more accurate and robust classification of mental states. Fusion strategies can be performed at the feature level, intermediate representation level, or decision level. Among these, decision-level fusion has advantages for heterogeneous signals such as EEG and EDA, since it allows each modality to be processed by architectures tailored to its characteristics, with their outputs combined at the prediction stage [20]. Decision-level fusion has been shown to improve recognition of stress and emotion across diverse physiological datasets, mitigating modality-specific weaknesses and reducing the risk of overfitting [21,22]. In construction noise research, this approach is especially relevant: impulsive sounds may be more salient in EEG activity, while tonal noise may manifest more strongly in EDA trends, making late fusion an effective strategy to capture the complementary strengths of each modality.

Most studies on noise and physiology have focused on transportation and general urban sources such as road traffic, railways, and aircraft, while construction-specific sounds have received much less attention. Mir et al. [23] reported that complex construction noises, such as those produced by saws and drills, disrupt cognitive and emotional states more strongly than steady sounds like bulldozers. In a related study, Mir et al. [24] showed that different types of construction noise significantly alter physiological responses, including heart rate, respiration, and EDA, underscoring the non-auditory health impacts of such exposures. Despite these findings, many investigations still rely on a single physiological channel or on handcrafted features, which often fail to capture the nonlinear and dynamic nature of human responses [20]. According to Lawhern et al. [19], conventional EEG analyses frequently depend on features tailored to specific tasks, which limit their generalizability. Multimodal deep learning approaches provide a way forward by enabling richer representations through the integration of complementary signals. Baltrušaitis et al. [22], who show that multimodal fusion typically outperforms unimodal methods, and from Hwang et al. [25], who demonstrate that combining EEG and EDA yields more accurate and nuanced stress assessments under construction noise than sound pressure level (SPL) measures alone. However, their approach relied on manually engineered features, tree-based models, and early fusion, which restrict scalability. Yang et al. [26] further show that joint multimodal modeling of physiological signals markedly improves stress recognition accuracy. These studies demonstrate the promise of multimodal methods but also make clear the gap and need for construction-focused, data-driven deep learning frameworks capable of capturing the complex and nonlinear physiological effects of noise exposure.

Our study addresses the gaps by building a deep learning-based framework that classifies annoyance caused by construction noise. We use CNNs to capture the fast-changing spatial and temporal features of EEG signals, and LSTM networks to model the slower, sequential patterns in EDA. We incorporated decision-level fusion to integrate complementary information from brain and autonomic responses. Annoyance levels are treated as a measurable indicator for cognitive and emotional stress. To go beyond a single baseline study, we also designed an extensive ablation study. By changing window lengths, training settings, fusion methods, and calibration strategies, we tested both the overall performance of the framework and the impact of each hyperparameter choice on its robustness and reliability. The importance of this study lies in three key contributions. First, we apply both single- and multimodal deep learning methods to the context of construction noise, which has received far less attention than traffic or other urban noise sources in physiological research. Second, our findings show that data-driven models can capture the complex and nonlinear dynamics of EEG and EDA signals under realistic noise conditions more effectively than traditional feature-based methods. Third, we provide a detailed ablation analysis that examines how different modeling choices, including unimodal and multimodal architectures, influence accuracy and stability. Fourth, we identify optimal hyperparameters via a comprehensive ablation study and re-evaluate the full pipeline under these settings, demonstrating how ablation-informed optimal hyperparameters improve overall performance and robustness. The outcome of this study deepens our understanding of how construction noise affects the brain and autonomic nervous system and indicates practical applications, such as real-time stress monitoring, safer construction site practices, and more informed noise-mitigation strategies in urban environments.

## 2. Materials and Methods

### 2.1. Laboratory Data Collection

**Figure 1:**
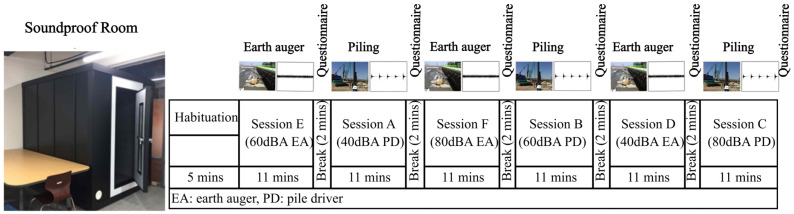
Experimental setup and protocol timeline [25].

We recruited 25 healthy volunteers (20 males and 5 females; mean age 24 years) with no reported hearing impairments or medical conditions. Before enrollment, participants were informed about the study aims and procedures and provided written consent. Hearing acuity was verified using a pure-tone audiogram [27], and trait noise sensitivity was assessed on a 0–10 scale [28]. The experiment consisted of six exposure sessions per participant, combining two classes of construction noise, impulsive noise generated by a pile driver and tonal noise produced by an earth auger, with three sound pressure levels (40, 60, and 80 dBA). The 40 dBA condition represented typical office background levels, 60 dBA corresponded to conversational or restaurant-like ambient noise, and 80 dBA approximated the intensity of common household equipment, such as a vacuum cleaner or lawnmower [25]. To safeguard participants, the maximum exposure level was restricted to 80 dBA, as prolonged exposure above 85 dBA may cause hearing damage. All sessions were conducted in a sound-insulated room designed to minimize external acoustic contamination and electrical interference. Participants were seated comfortably and instructed to minimize head and facial movements. To ensure adaptation, each participant was given five minutes to habituate to the environment and EEG apparatus prior to the first session. Each exposure session lasted approximately 11 min and was separated by a 2 min break to reduce fatigue and stabilize physiological baselines. After every session, participants completed an ISO-standardized annoyance questionnaire (0–10 scale) to report their subjective stress responses. Several measures were taken to control potential confounding factors. The experiment was conducted under consistent environmental conditions, with stable temperature and humidity, and sessions were scheduled at the same time of day to minimize circadian effects. The experimental setting was standardized without visual stimuli to reduce perceptual distractions, and participants’ health conditions were confirmed prior to data collection. To ensure the consistency and accuracy of SPL measurements across different recording and playback environments, a sound calibrator generating a 1 kHz pure tone at 94 dB SPL was employed as a reference. This calibration was performed prior to field recordings and during laboratory experiments involving noise playback, allowing all SPL values to be uniformly adjusted based on the same standard reference. To limit learning and order effects, the sequence of noise conditions was randomized for each participant following the protocol of Ref. [25]. The overall study design is summarized in Figure 1.

**Figure 2:**
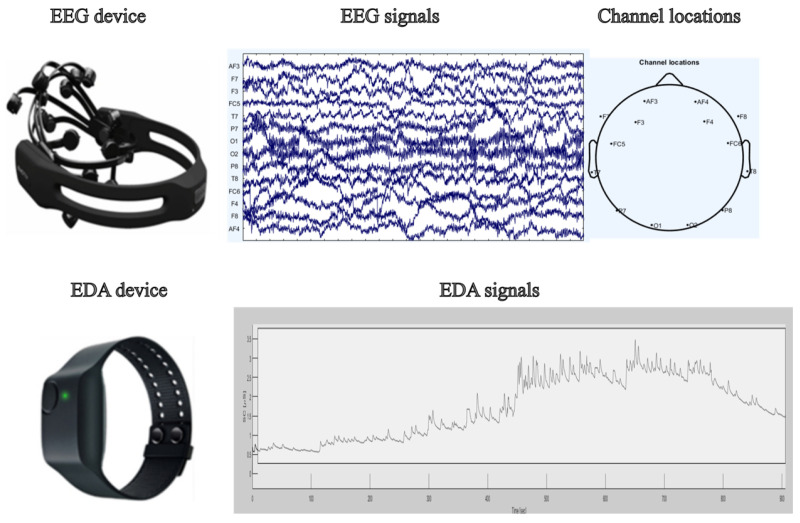
Data collection device and sample signals.

**Figure 3:**
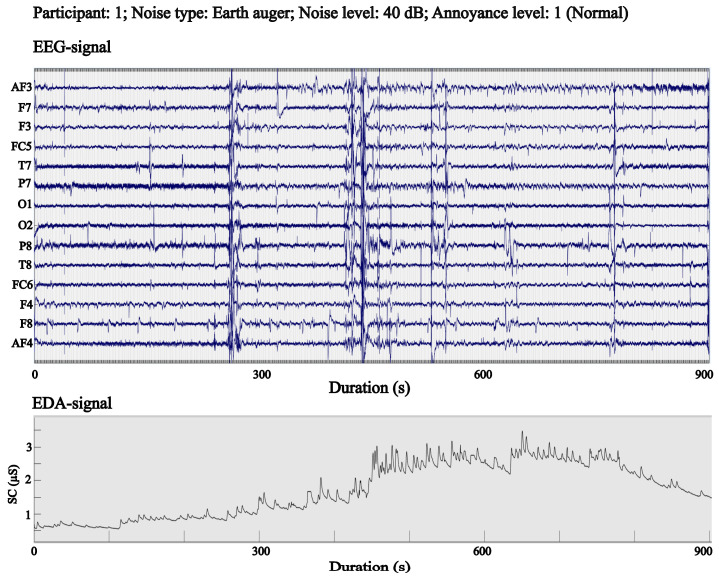
Illustration of concurrently recorded EEG and EDA signals for a participant.

EEG was recorded with a wearable *Emotiv EPOC+* system at 128 Hz from 14 scalp sites: AF3, AF4, F3, F4, F7, F8, FC5, FC6, O1, O2, P7, P8, T7, and T8 as shown in Figure 2. The montage included two reference electrodes, a common-mode sense electrode on the left mastoid and a driven right-leg electrode on the right mastoid, which improve rejection of common-mode noise and enhance signal stability [29]. EDA was recorded concurrently using an *Empatica E4* wrist device at a sampling rate of 4 Hz (Figure 2). The resulting dataset consists of time-aligned EEG and EDA streams for each of the six noise conditions per participant. Each participant completed six sessions, one for each combination of noise type and SPL. During each session, the target noise was presented while EEG and EDA were continuously recorded. The order of sessions was organized to maintain comparable exposure durations across participants and to reduce potential order effects. Data quality was monitored during and after acquisition. Runs with marked motion artifacts, persistent electrode contact issues, or prolonged wireless dropouts were flagged. Participants with excessive contamination across multiple sessions were excluded from analysis, which led to the removal of five participants, as noted above. For the retained sample, EEG and EDA streams were visually inspected and exported in standardized formats (EEGLAB. set for EEG and CSV for EDA) for downstream processing. The three SPLs span a range from typical indoor background conditions to levels that are common near active equipment. This range allows for an analysis of physiological responses to exposure to noise levels ranging from minimally intrusive to clearly noticeable, and it enables a comparison of impulsive and tonal noise classes across comparable intensities. Figure 3 presents representative EEG and EDA recordings from a single participant, annotated with the corresponding annoyance level.

### 2.2. Data Processing, Deep Learning, and Ablation Studies

**Figure 4:**
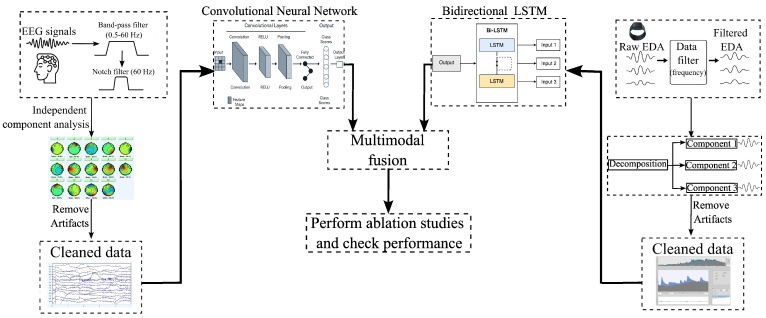
Flowchart of the framework.

Figure 4 demonstrates the framework of the research. The proposed framework integrates EEG and EDA signals for multimodal analysis. EEG was preprocessed in EEGLAB using zero-phase, Hamming-window FIR filters (*pop_eegfiltnew*). Signals were band-pass filtered at 0.5–60 Hz and additionally notch-filtered at 60 Hz (stopband 58–62 Hz). Filter order was automatically selected by EEGLAB based on sampling rate and transition width; the design is zero-phase (forward–backward) with no latency distortion. After filtering, data were average-re-referenced and decomposed by independent component analysis (ICA), and non-brain components were removed. In parallel, EDA was sampled at 4 Hz and denoised with a 4th-order Butterworth low-pass filter (cutoff = 1.9 Hz) applied in zero-phase. We derived a tonic–phasic decomposition using a 10 s centered moving average for the tonic level and defined the phasic component as the residual. The preprocessed EEG signals were passed through a CNN, which captures spatial and spectral patterns, while the EDA signals were processed with a bidirectional LSTM (Bi-LSTM) network to model temporal dependencies in the physiological responses. The outputs of these networks were combined through multimodal fusion to leverage complementary information from both modalities. Finally, we conducted ablation studies to examine how different parameters influence deep learning behavior and model performance. Performance metrics were systematically computed to evaluate the effectiveness of the integrated framework.

#### 2.2.1. EEG and EDA Data Processing

EEG signals were preprocessed in EEGLAB [30]. Because scalp EEG is sensitive to ocular, muscular, and environmental artifacts [31], we applied a band-pass filter of 0.5–60 Hz to remove slow drift and high-frequency noise, followed by a 60 Hz notch filter to suppress power-line interference [32]. Channels with poor signal quality were detected using a combination of flatline duration, abnormal variance, and low correlation with neighbors. Marked channels were replaced by spherical spline interpolation to preserve spatial structure. We then ran ICA with *runica* to separate artifact sources. Components reflecting blinks, saccades, and scalp muscle activity were identified by their time courses, spectra, and scalp maps, and were removed prior to back-projection to the sensor level. The data were inspected visually after each step to verify signal integrity. Raw EDA from the *Empatica E4* was first low-pass filtered to suppress transient high-frequency contamination and outliers. Short segments contaminated by motion or non-physiological spikes were flagged and linearly interpolated if brief; longer segments were excluded. We applied continuous decomposition analysis (CDA) to separate tonic skin conductance level from phasic responses and to estimate the underlying sudomotor nerve activity driver [33]. This representation reduces the influence of slow baseline drift and isolates stimulus-locked arousal dynamics that are most informative for classification.

We developed a deep learning framework that learns from EEG and EDA jointly to classify annoyance (normal vs. high) during exposure to construction noise. Annoyance was originally rated on a 10-point scale, where 1 denotes the lowest annoyance and 10 the highest. In this study, we binarized labels:

(1) $$y=\left\{\begin{array}{c} 0\;if\;annoyance\;level\leq 7,\\ 1\;if\;annoyance\;level>7 \end{array}\right.$$

In prior work, Hwang et al. [25] defined “high” annoyance as ratings greater than 8, with all other values treated as “others”. In contrast, our study adopted a slightly broader threshold, classifying annoyance levels greater than 7 as “high” and those less than or equal to 7 as “normal”. After preprocessing and alignment, each session was segmented into non-overlapping windows. EEG windows ranged from 1 to 8 s and EDA windows from 5 to 30 s in ablation (baseline: 2 s EEG, 20 s EDA). For each fold, normalization statistics (per-channel mean and standard deviation) were computed on the training split only and applied to validation and test sets. The model operates directly on time-domain signals so that features are learned end-to-end, rather than engineered.

#### 2.2.2. EEG Deep Learning Model

EEG was sampled at 128 Hz. For a window length of $W_{EEG}$ seconds, each input segment had the following shape:

(2) $$X\in R^{C\times T},\;T=128\;\cdot \;W_{EEG}$$

where C is the number of channels and T is the number of samples per channel in the window. To suppress baseline drift, the per-channel mean was removed. Each channel was then standardized by subtracting the training mean and dividing by the training standard deviation. Each channel was detrended by subtracting its mean and standardized using training statistics.

We employed a compact CNN designed to capture both short-lived transients and interactions across channels. Convolutions are particularly suited for EEG because they act as adaptive filters that can learn frequency-specific or spatiotemporal patterns that are difficult to predefine. The CNN consisted of four convolutional blocks, with filter counts increasing from 32 to 256. Each block applied a 3×3 convolution, followed by batch normalization, a rectified linear unit (ReLU) activation, and temporal max pooling. This progressively enlarged the temporal receptive field, allowing the network to integrate information across millisecond-to-seconds timescales while attenuating high-frequency noise.

Formally, the transformation of block i is written as follows:

(3) $$X_{i}=\sigma (BN_{i}\left({Conv}_{3\times 3}^{F_{i-1}\to F_{i}}\left(Pool\left(X_{i-1}\right)\right)\right))$$

where $F_{i}\in \left\{32, 64, 128,\left.256\right\}\right.,\;\sigma$ is ReLU, BN is batch normalization, and Pool is temporal max-pooling.

An adaptive average pooling layer aggregated the final feature maps into a fixed-length 256-dimensional vector, independent of the input window size. A small multilayer perception (MLP) was then applied, with a hidden layer of 128 units and dropout regularization. Finally, a linear classifier produced two output logits:

(4) $$z_{e}=W_{2}\sigma \left(W_{1}X_{4}+b_{1}\right)+\;b_{2}$$

These logits represent the uncalibrated evidence for the two annoyance classes. Dropout with probability 0.5 was applied between fully connected layers to reduce overfitting. This architecture reflects the intuition that annoyance-related EEG patterns may appear as transient bursts or spatial synchronizations, both of which CNNs are well suited to capture.

#### 2.2.3. EDA Deep Learning Model

EDA was sampled at 4 Hz, a much lower sampling rate than EEG, reflecting its slower physiological dynamics. For a window length of $W_{EDA}$ seconds, each input segment had the following shape:

(5) $$x_{1:T}\in R^{T},\;T=4\;\cdot \;W_{EDA}$$

As with EEG, the mean was removed, and each window was standardized using training statistics.

We modeled EDA with a two-layer BiLSTM network. This choice was motivated by the autocorrelated nature of EDA, which contains both slow tonic shifts and rapid phasic responses. Recurrent units can integrate information across long horizons and are therefore a natural fit. At each time step, t the forward and backward LSTM states were updated as follows:

(6) $${\vec{h}}_{t},{\vec{c}}_{t}={LSTM}_{f}\left(x_{t},{\vec{h}}_{t-1},{\vec{c}}_{t-1}\right),\;{\overset{⃐}{h}}_{t},{\overset{⃐}{c}}_{t}={LSTM}_{f}\left(x_{t},{\overset{⃐}{h}}_{t+1},{\overset{⃐}{c}}_{t+1}\right)$$

The final hidden representation was formed by concatenating the last forward and backward hidden states:

(7) $$h=\left[{\vec{h}}_{T};{\overset{⃐}{h}}_{1}\right]\in R^{128}$$

This representation was passed through a small MLP (128 → 64 units with dropout), followed by a linear output layer:

(8) $$z_{d}=\;W_{2}\sigma \left(W_{1}h+\;b_{1}\right)+\;b_{2},\;z_{d}\in R^{2}$$

Here, $z_{d}$ represents the EDA logits for normal vs. high annoyance.

The bidirectional encoder summarized both long-term tonic activity and short-term phasic responses. This dual capacity is critical because both sustained arousal and brief spikes in EDA can signal changes in perceived annoyance.

All models were implemented in PyTorch (version 2.7.1 with CUDA 12.8). Training defaults were batch size 64, learning rate 10^−3^, weight decay 10^−4^, and up to 100 epochs with early stopping (patience 50). *AdamW* was used as an optimizer. *ReduceLROnPlateau* adjusted the learning rate adaptively, and mixed precision training was enabled when supported by hardware. Class imbalance was handled by weighting the cross-entropy loss according to training label frequencies. Data augmentation was evaluated in ablation: training windows were duplicated up to 10 times with additive Gaussian noise (standard deviation up to 0.10). Importantly, augmentation was disabled for validation and test sets.

#### 2.2.4. Fusion of EEG and EDA

Before fusion, each model’s output logits were calibrated using temperature scaling on a held-out validation subset. Given logits z, the following calibration applies:

(9) P=softmax(zt^)

where $\hat{t}$ is a learned temperature optimized to minimize cross-entropy on validation data. Calibration improves the interpretability of probabilities, ensuring that decision thresholds and fusion are based on well-calibrated confidence scores rather than arbitrary logit magnitudes. In this study, we investigated three late-fusion strategies: stacked linear fusion, weighted averaging, and gated fusion. Fusion was employed in a conservative manner; it was applied exclusively at test time and only when it demonstrated superior performance over the EEG branch alone in terms of both accuracy and F1 on the validation subset.

#### 2.2.5. Ablation Studies

To examine the contribution of design choices, we conducted 44 ablation experiments. Each ablation varied one factor while keeping all others fixed to the baseline configuration. Table 1 organizes the ablation protocol into parameter groups, each targeting a specific design choice that could plausibly affect model performance. The first group of experiments examined fusion strategy, comparing stacked linear fusion, weighted averaging, and gated fusion against unimodal baselines (EEG-only and EDA-only) to determine the added value of multimodal learning. The second set of experiments varied elements of the training regime, including batch size, learning rate, and patience, to capture the sensitivity of optimization to stability and convergence. Weight decay and L1 penalty were also manipulated to evaluate the role of explicit regularization.

Windowing strategy formed another major axis of ablation. Because the temporal granularity of physiological signals is not fixed, EEG window lengths were tested between 1 and 8 s and EDA between 5 and 30 s. This allowed us to prove how temporal segmentation provided stronger support for annoyance classification. Data scarcity motivated a further set of experiments focused on augmentation and noise injection, where different levels of offline window duplication were introduced to test whether synthetic variability enhanced generalization. Finally, the validation protocol itself was varied, with the fraction of training data held out for validation ranging from 10% to 25%, and patience for early stopping varied between 15, 25, and 40 epochs to test effects on stability.

## 3. Results and Discussions

This section presents the results of annoyance classification using EEG and EDA signals with single- and multi-modal deep learning approaches, followed by analyses of factors influencing model performance. We first describe the dataset characteristics and baseline performance, then examine the contributions of individual design choices through a series of ablation studies. The findings depict the impact of fusion strategy, windowing, training dynamics, regularization, augmentation, and validation design on classification outcomes.

### 3.1. Dataset and Class Distribution

The dataset used in this study consisted of synchronized EEG and EDA recordings collected under controlled construction-noise exposure conditions. Each trial was labeled with an annoyance score ranging from 1 (no annoyance) to 10 (extreme annoyance), subsequently binarized into normal annoyance (1–7) and high annoyance (8–10) categories. This binarization was guided by prior research showing that annoyance scores above 7 are strongly associated with heightened stress responses and negative affective states (reference). A total of 5013 EEG–EDA segments were extracted after preprocessing and windowing. Following segmentation, the dataset exhibited class imbalance, with 3356 segments belonging to the low-annoyance class and 1657 segments to the high-annoyance class, corresponding to a ratio of approximately (67:33). The imbalance reflects natural tendencies in environmental noise perception, where mild or moderate annoyance ratings are more frequently reported than extreme annoyance. To mitigate potential bias from this skewed distribution, several measures were adopted. First, mean metrics (precision, recall, F1) were used in all evaluations to ensure that both classes contributed equally to performance assessment. Second, class-balanced loss functions were applied during training to reduce overfitting toward the majority class. Finally, augmentation strategies were incorporated, including duplication of minority-class samples and Gaussian noise injection, to balance effective training exposure.

The class distribution is summarized in Table 2. The confusion matrices presented in later sections also highlight how this imbalance influenced classification, with misclassifications occurring more frequently in the high-annoyance category. Table 3 summarizes each noise type (earth auger, pile driver) and tonal condition (40/60/80 Hz) for which the 25 participants reported normal vs. high annoyance. Two consistent patterns emerge. First, annoyance rises with frequency: for earth auger, only 1/25 subjects reported high annoyance at 40 Hz and 5/25 at 60 Hz, but 20/25 at 80 Hz; for the pile driver, the counts were 2/25, 4/25, and 17/25, respectively. Second, there is strong inter-subject variability, i.e., the same individuals do not always cluster in the same annoyance category across conditions. This distribution demonstrates the class imbalance found in our windowed datasets.

### 3.2. Baseline Performance

The baseline system combined a CNN for EEG with a Bi-LSTM for EDA, integrated through stacked late fusion and a validation-gated fallback to EEG (Table 3). EEG was segmented into non-overlapping 2 s windows (128 Hz) and EDA into non-overlapping 20 s windows (4 Hz). We evaluated using 5-fold subject-independent cross-validation (GroupKFold): all windows from a given participant were assigned to a single fold to prevent leakage. For each training fold, 15% of the training windows formed a validation set; this set was split 1:1 into a calibration subset (for temperature scaling) and a fusion/threshold subset (for F1-optimal thresholding and deciding between stacked fusion and EEG). On each fold, fusion was used on the test set only if it outperformed EEG in terms of both accuracy and F1 on the fusion-validation subset; otherwise, predictions defaulted to EEG. Test folds were not used for training, calibration, thresholding, or model choice.

In Table 4, with 2 s EEG windows, 20 s EDA windows, a batch size of 64, temperature scaling, and post hoc thresholding on calibrated scores, the fused model reached an accuracy of 0.796 (95% CI: 0.769–0.823), macro-F1 of 0.766 (0.733–0.798), and AUROC of 0.856 (0.819–0.892). The fold-to-fold coefficient of variation (CoV) remained low (≤5% across metrics), indicating stable generalization. EEG alone performed similarly (accuracy 0.794 [0.768–0.820], F1 0.765 [0.733–0.797]) and showed comparably low CoV (e.g., 7.07% for accuracy, 3.86% for F1), indicating high stability across splits. EDA alone remained substantially weaker (accuracy 0.557 [0.528–0.586], F1 0.539 [0.517–0.561]), despite relatively low CoV, confirming that the performance gap is systematic and not due to high variance. Because prior work (Hwang et al. [25]) used a different annoyance label definition and an early-fusion architecture, we initially treated their results as contextual rather than directly comparable. To enable a fairer comparison, we re-ran our models using the same annoyance cutoff as Hwang et al. [25] and report the aligned results in Table 5. Under matched conditions, our approach outperforms that of Ref. [25] in both unimodal EEG (accuracy 0.834 vs. 0.6383; F1 0.771 vs. 0.5892) and multimodal fusion (accuracy 0.846 vs. 0.6517; F1 0.775 vs. 0.5967), indicating that our late-fusion strategy is more effective than their early fusion for annoyance classification.

### 3.3. Ablation Study

To better understand the contribution of individual design choices to deep learning model performance, we conducted a systematic ablation study. Each group of parameters was varied independently while keeping the others fixed, allowing us to evaluate the effect of architectural, training, and preprocessing factors. In total, 44 different settings were explored across nine parameter groups. The ablation study includes fusion strategy, window size, batch size, training dynamics, regularization, augmentation factor, and validation fraction. The performance metrics were evaluated after multimodal fusion, rather than focusing on the individual performance of EEG and EDA modalities, as the focus of the ablation studies is on the effectiveness of the integrated framework.

#### 3.3.1. Fusion Strategy Comparison

Table 6 shows that EDA-only obtained a noticeably weaker performance (accuracy 0.56, F1 0.539, AUROC 0.56) than EEG-only, which produced the strongest unimodal performance (accuracy 0.794, F1 0.761, AUROC 0.84). Fusion models are typically more stable over the folds and approach or marginally surpass EEG in mean performance when the two modalities are merged. Specifically, the stacked fusion model achieved the lowest coefficient of variation (CoV ≤ 3% for all metrics, compared with 7–8% for EEG), the greatest accuracy (0.804), and the competitive F1 (0.77) and AUROC (0.842). Gated fusion performed marginally worse than EEG, but it is still more stable (accuracy CoV 4.02% vs. 7.17%). These results indicate that multimodal fusion provides comparable or better accuracy than EEG alone while improving robustness and reducing variability across folds.

#### 3.3.2. Sensitivity to Windowing

**Figure 5:**
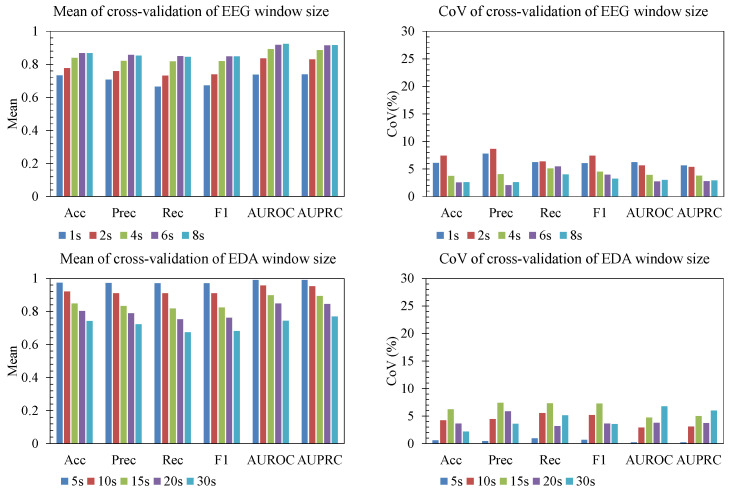
Window size effect.

Figure 5 demonstrates the effect of window size on both EEG and EDA signals. We report both the mean and the coefficient of variation (CoV) of all performance metrics across folds to characterize accuracy and stability. Window size had a clear impact on performance, and the effect differed by modality. For EEG, medium window size (6 s) yielded the highest performance, with accuracy and F1 peaking in this range. Performance declined for longer windows (8 s), likely because temporal averaging over longer segments attenuates fast neural responses to noise. For EDA, the pattern was different: shorter segments (5 s) produced the strongest results in this dataset (e.g., accuracy 0.97, F1 0.97, AUROC 0.99), whereas performance dropped as the window increased, with 20–30 s windows showing substantially lower accuracy and F1. This indicates that temporal resolution should be matched to the intrinsic dynamics of each modality: EEG benefits from short windows that preserve rapid fluctuations, while EDA typically benefits from slower integration but still degrades if windows become too long and the number of training samples drops.

The CoV analysis further supports these findings. Variability across folds remained generally low (mostly <8%) for all EEG window sizes and metrics, indicating consistent generalization rather than fold-specific effects. Slightly elevated CoV for precision and F1 at the shortest EEG windows (1–2 s) suggests modest fold-to-fold fluctuation in those cases, while longer windows (4–8 s) showed a lower CoV for accuracy, F1, AUROC, and area under the precision–recall curve (AUPRC), indicating more stable behavior. These trends suggest that the reported performance differences are systematic, not driven by a single fold.

#### 3.3.3. Training Dynamics and Regularization

**Figure 6:**
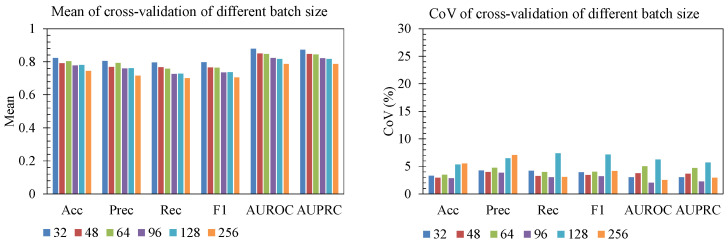
Batch size effect.

**Figure 7:**
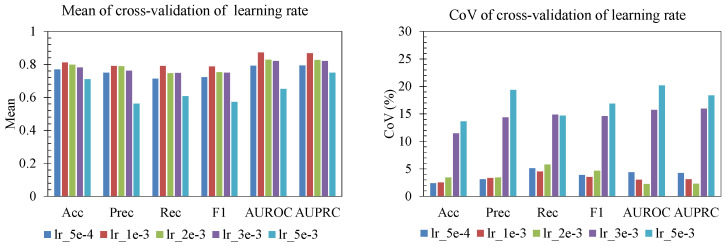
Effect of learning rate.

**Figure 8:**
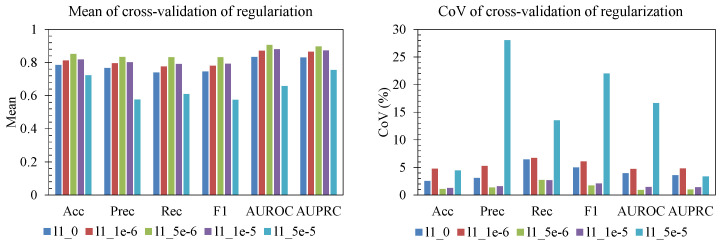
Effect of regularization.

The ablation study showed that performance and stability were both sensitive to batch size, learning rate, and L1 regularization (Figure 6, Figure 7 and Figure 8). For batch size (Figure 6), smaller batches (32–64) produced the highest mean accuracy, precision, recall, F1, AUROC, and AUPRC. As batch size increased beyond 96, performance declined and became less reliable, with batch 256 showing both lower mean scores and elevated CoV for several metrics. This indicates that overly large batches hurt generalization. For learning rate (Figure 7), rates near 1 × 10^−3^ and 2 × 10^−3^ achieved strong mean performance with a relatively low CoV across folds, whereas too-small learning rates (5 × 10^−4^) underperformed, and aggressive rates (≥3 × 10^−3^) led to large fold-to-fold variability (CoV > 10–20% for AUROC/AUPRC), consistent with unstable convergence. Finally, for L1 regularization strength (Figure 8), mild penalties (10^−6^–5 × 10^−6^) led to higher mean F1, AUROC, and AUPRC with low CoV (<5%), while stronger L1 (≥10^−5^) sharply reduced accuracy/F1 and produced high variability (CoV > 15–20%). The most reliable region is characterized by batch size 32–64, learning rate on the order of 10^−3^, and weak L1 regularization, which jointly yield high mean performance and low CoV, indicating both good accuracy and stable generalization across folds.

#### 3.3.4. Data Augmentation and Weight Decay

**Figure 9:**
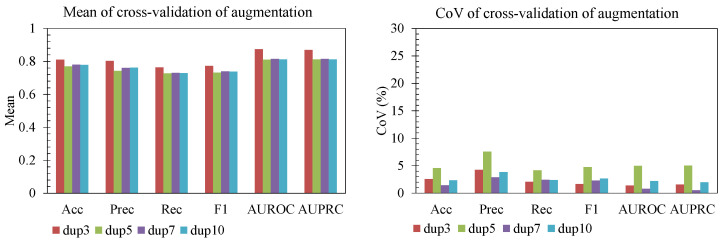
Effect of augmentation.

**Figure 10:**
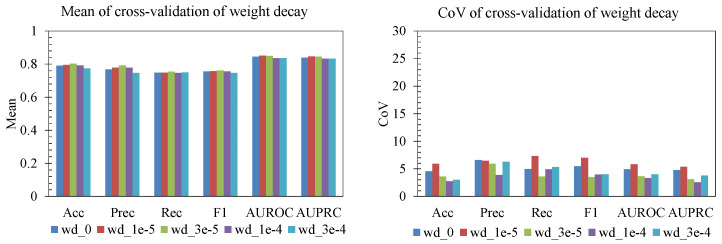
Effect of weight decay.

We further examined the effects of data augmentation and weight decay on both mean performance and stability across folds (Figure 9 and Figure 10). For augmentation, a duplication factor of 3 (dup3) achieved slightly higher accuracy, precision, recall, and F1 than heavier duplication (dup5–dup10). At the same time, the coefficient of variation (CoV) remained low (generally <5%), indicating that these gains were consistent and not driven by a single fold. Increasing duplication beyond 3 did not yield additional improvement and in some cases increased variability (e.g., precision and recall for dup7), suggesting diminishing returns once synthetic balance saturates. For weight decay, moderate values (3 × 10^−5^ to 1 × 10^−4^) produced the best trade-off: a high AUROC/AUPRC and strong F1, with a relatively low CoV across folds. With no decay, performance and stability both degraded slightly, while overly strong decay (3 × 10^−4^) reduced accuracy and F1 and increased variability. Hence, mild augmentation (dup3) and moderate weight decay (10^−4^) improve generalization while keeping variance across folds controlled.

#### 3.3.5. Validation Fraction

**Figure 11:**
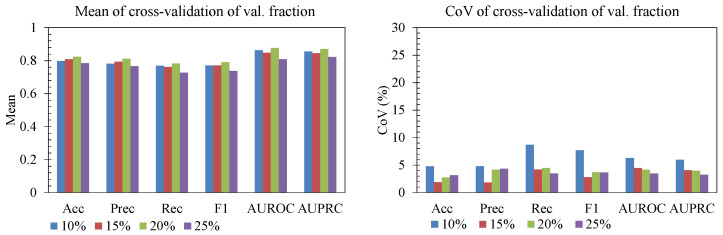
Effect of validation fraction.

Figure 11 shows how different validation split sizes (10%, 15%, 20%, 25%) affect both mean performance and stability across folds. Mean accuracy, AUROC, and AUPRC generally increased as the validation fraction grew from 10% to 20%, and then plateaued, while recall and F1 did not substantially improve beyond 20%. The coefficient of variation (CoV) was highest at 10%, indicating unstable fold-to-fold behavior when the validation set is too small to tune thresholds and fusion. Splits of 20–25% produced both high mean performance and a low CoV, although at 25% we observed a slight reduction in recall and F1, consistent with having less data left for training. Allocating about 20% of the data to validation provided the best trade-off between reliable validation signals and preserving training capacity.

### 3.4. Ablation-Informed Optimal Model Performance

We selected the best performing hyperparameters from the ablation study, as shown in Table 7, and re-ran the full subject-independent 5-fold evaluation. The architecture mirrors our baseline, such as CNN for EEG and a Bi-LSTM for EDA with stacked late fusion, while adopting the ablation-derived settings: batch size 32, learning rate 0.001, EEG windows of 6 s, EDA windows of 5 s, weight decay $3\times 10^{-5}$, L1 $5\times 10^{-6}$, duplication-based augmentation k=3, and a 20% validation split. As in the main pipeline, fusion on the test fold was used only when it exceeded EEG on both accuracy and F1 on the held-out fusion-validation subset; otherwise, predictions defaulted to EEG. No test data were used for calibration, thresholding, or model selection. Fold-mean results with 95% CIs are reported in Table 8.

Table 7 summarizes performance after re-training with the ablation-informed optimal hyperparameters. EEG remains the dominant modality, with 0.90 accuracy and 0.887 F1, tight 95% CIs, and very high separability (AUROC: 0.95; AUPRC: 0.943). EDA alone is markedly weaker across all metrics, consistent with our earlier findings. The fused model closely tracks EEG, with only marginal, non-material differences (e.g., accuracy 0.903 vs. 0.900; F1 0.888 vs. 0.887), and the overlapping confidence intervals indicate no clear superiority. The narrow confidence intervals indicate stable performance across folds, and the strong AUROC/AUPRC values suggest reliable ranking even when operating points change. The ablation-tuned setup improves EEG a bit, keeps fusion on par with it, and shows that EDA helps only occasionally. Compared to Table 4 (baseline), the ablation-optimized setup in Table 7 substantially lifts EEG and fusion, e.g., accuracy rises from 0.79 to 0.90 (EEG) and 0.80 to 0.90 (Fusion), with AUROC from 0.86 to 0.95 for both. EDA remains weak, showing only a marginal uptick (accuracy: 0.56–0.57) and still lagging far behind EEG.

## 4. Conclusions and Recommendations

This study investigated single- and multimodal deep learning approaches for annoyance classification using EEG and EDA signals recorded under controlled noise-exposure conditions. The results confirmed that neural networks are effective for extracting discriminative features from both modalities, with EEG contributing the strongest signal and EDA offering complementary information that improved calibration and stability. The baseline model, combining a CNN for EEG and a Bi-LSTM for EDA, achieved robust performance, and the ablation studies provided a systematic understanding of how architectural and training choices shape outcomes. The following are the key findings of this research:

- CNN effectively captured transient neural responses from short-duration EEG segments, while the LSTM model leveraged slower autonomic trends in EDA, indicating that the two modalities encode complementary aspects of annoyance-related physiological state.
- Model performance depended on matching the analysis window to each modality’s temporal dynamics: for EEG, a 6 s window provided a strong balance between high mean performance and low fold-to-fold variability (low CoV), while for EDA, 5 s were more effective. This highlights the need to co-design annotation granularity, window length, and network architecture.
- Smaller batch sizes (32–64) and learning rates on the order of 10^−3^ produced both high accuracy and low cross-validation variability. Moderate weight decay (10^−4^) further improved AUROC and AUPRC, whereas more aggressive regularization or higher learning rates degraded stability. L1 penalties provided limited additional benefit.
- Augmentation improved class balance slightly but exhibited diminishing returns at higher duplication levels, suggesting that simple resampling is not the primary driver of robustness.
- Varying the validation fraction between 10% and 25% produced comparable accuracy, F1, and calibration, with a 20% split offering a practical balance between reliable model selection and sufficient training capacity.
- EEG alone remained the strongest unimodal predictor, whereas EDA alone performed notably worse. Nonetheless, fusion (particularly stacked fusion) matched or slightly exceeded EEG in mean accuracy, F1, and AUROC while reducing fold-to-fold variability. This indicates that integrating EEG and EDA improves reliability across subjects, even when one modality is comparatively weak.
- With the ablation-informed hyperparameters, both EEG and the fused model improved substantially over the baseline (accuracy: 0.80–0.90; AUROC: 0.86–0.95) and showed tight confidence intervals, indicating stable generalization.

These findings demonstrate that deep learning is a useful framework for annoyance classification if models are carefully aligned with the temporal and statistical properties of the data and supported by appropriate regularization and training strategies. The current results also surpass those reported in previous studies, as noted earlier. However, several limitations remain. The dataset was moderately imbalanced, reducing recall for high-annoyance states. The experiments were conducted under controlled laboratory conditions, which may not fully represent the variability of real-world construction environments. This study used 5-fold cross-validation without subject-wise separation. Therefore, the results reflect overall performance rather than strict subject-independent generalization. In addition, all participants were healthy and within the 20–30-year age range. Augmentation was limited to simple duplication and Gaussian noise, leaving more advanced strategies unexplored. Furthermore, participants reported annoyance once after 11 min sessions, which cannot capture within-session fluctuations and may introduce recall or accumulation bias. This likely makes our estimates conservative and can blur short-lived responses.

Future work should develop imbalance-aware deep learning methods, explore adaptive architectures for temporal resolution, and validate models on larger datasets from real construction settings. Such efforts will enhance the reliability and applicability of annoyance detection for occupational safety, noise assessment, and human–machine interaction.

## Figures and Tables

**Table 1:** Overview of ablation experiments.

Parameter Group	Settings	Runs
Fusion strategy	Stacked, weighted, gated, EEG-only, EDA-only	5
Batch size	32, 48, 64, 96, 128, 256	6
Learning rate	5 × 10^−4^, 1 × 10^−3^, 2 × 10^−3^, 3 × 10^−3^, 5 × 10^−3^	5
EEG window length	1, 2, 4, 6, 8 s	5
EDA window length	5, 10, 15, 20, 30 s	5
Weight decay	0, 1 × 10^−5^, 3 × 10^−5^, 1 × 10^−4^, 3 × 10^−4^	5
L1 penalty	0, 1 × 10^−6^, 5 × 10^−6^, 1 × 10^−5^, 5 × 10^−5^	5
Augmentation factor	3, 5, 7, 10 duplications (with Gaussian noise)	4
Validation fraction	0.10, 0.15, 0.20, 0.25 of training data	4
Total		44

**Table 2:** Class distribution.

Class	Number of Segments	Percentage
Normal annoyance	3356	67
High annoyance	1657	33

**Table 3:** Annoyance classes among participants.

Noise Type	Noise Level	Normal Annoyance	High Annoyance
Earth auger	40 Hz	1–9, 11–25	10
	60 Hz	1–9, 11–14, 17–22, 24	10, 15, 16, 23, 25
	80 Hz	3, 8, 19–21	1–2, 4–7, 9–18, 22–25
Pile driver	40 Hz	1–9, 11–22, 24–25	10, 23
	60 Hz	1–8, 11–13, 15–22, 24–25	9–10, 14, 23
	80 Hz	3, 6–7, 13–14, 16–18	1–2, 4–5, 8–12, 15, 19–25

**Table 4:** Performance metrices of baseline conditions.

Model		Accuracy	Precision	Recall	F1	AUROC	AUPRC
EEG	Mean	0.794	0.772	0.765	0.765	0.856	0.851
	CoV (%)	7.07	3.23	5.49	3.86	2.93	2.52
	95% CI	0.768, 0.820	0.739, 0.805	0.722, 0.808	0.733, 0.797	0.817, 0.895	0.816, 0.885
EDA	Mean	0.557	0.549	0.555	0.539	0.555	0.532
	CoV (%)	5.34	3.65	4.32	2.64	1.61	0.90
	95% CI	0.528, 0.586	0.530, 0.568	0.533, 0.577	0.517, 0.561	0.541, 0.568	0.522, 0.542
Fusion	Mean	0.796	0.775	0.764	0.766	0.856	0.85
	CoV (%)	2.44	3.23	5.49	3.86	2.93	2.52
	95% CI	0.769, 0.823	0.739, 0.811	0.721, 0.807	0.733, 0.798	0.819, 0.892	0.817, 0.883

Hyperparameters under baseline conditions: fusion strategy: stacked; batch size: 64; learning rate: 0.001; window size (EEG): 2 s; window size (EDA): 20 s; weighted decay: 0.0001; L1-regularization: 0; duplication (augmentation): 5; validation fraction: 0.15.

**Table 5:** Performance metrices and comparison with Hwang et al., 2025 [25].

Model	EEG		Fusion	
	This Study	Hwang et al. [25]	This Study	Hwang et al. [25]
Accuracy	0.834	0.6383	0.846	0.6517
F1	0.771	0.5892	0.775	0.5967

**Table 6:** Performance metrices of different fusion strategies.

Fusion Mode	Accuracy Mean, CoV (%)	F1 Mean, CoV (%)	AUROC Mean, CoV (%)
EDA only	0.56, 5.34	0.539, 2.64	0.56, 1.61
EEG only	0.794, 7.17	0.761, 7.60	0.84, 4.81
Gated	0.78, 4.02	0.738, 4.85	0.823, 5.53
Stacked	0.804, 2.39	0.77, 3.86	0.842, 2.93
Weighted	0.74, 5.16	0.68, 5.25	0.74, 5.12

**Table 7:** Optimal hyperparameters from the ablation study.

Hyperparameter	Value/Method
Fusion strategy	Stacked
Batch size	32
Learning rate	0.001
Window size (EEG)	6 s
Window size (EDA)	5 s
Weighted decay	3 × 10^−5^
L1-regularization	5 × 10^−6^
Duplication (augmentation)	3
Validation fraction	0.20

**Table 8:** Performance metrices with ablation-informed optimal hyperparameters.

Model	EEG		EDA		Fusion	
	Score	95% CI	Score	95% CI	Score	95% CI
Accuracy	0.90	0.872, 0.928	0.568	0.540, 0.595	0.903	0.875, 0.930
Precision	0.891	0.857, 0.924	0.555	0.542, 0.569	0.892	0.859, 0.926
Recall	0.886	0.850, 0.922	0.561	0.546, 0.576	0.885	0.853, 0.916
F1	0.887	0.855, 0.920	0.547	0.529, 0.565	0.888	0.857, 0.918
AUROC	0.95	0.934, 0.966	0.566	0.553, 0.579	0.950	0.934, 0.965
AUPRC	0.943	0.924, 0.962	0.543	0.535, 0.550	0.943	0.924, 0.962

## Data Availability

The raw data supporting the conclusions of this article will be made available by the authors on request due to privacy and ethical restrictions. The dataset contains individual participants’ biosensing data (e.g., EEG, EDA), which are considered sensitive personal information. However, de-identified datasets may be available from the corresponding author upon reasonable request.
